# Unmasking Factor XIII Deficiency: Recurrent Intracranial Hemorrhage Despite Normal Coagulation Studies

**DOI:** 10.7759/cureus.113621

**Published:** 2026-07-29

**Authors:** Karthik Ramesh, Gavin Hui, Michael Shodiya, Nicolaos J Palaskas

**Affiliations:** 1 Department of Medicine, University of California, Los Angeles, Los Angeles, USA; 2 Department of Medicine, Division of Hematology and Oncology, University of California, Los Angeles, Los Angeles, USA

**Keywords:** bleeding disorders, congenital coagulopathy, congenital factor xiii deficiency, cryoprecipitate, intracranial hemorrhage (ich)

## Abstract

Factor XIII (FXIII) deficiency is a rare bleeding disorder that can be easily missed because routine coagulation tests remain normal. We report a 32-year-old Nicaraguan man who carried a childhood diagnosis of von Willebrand disease and was inadvertently managed with cryoprecipitate for 25 years, a treatment that incidentally corrected his true underlying deficiency due to its high FXIII content. After immigrating and transitioning to von Willebrand factor/Factor VIII concentrate, he developed recurrent life-threatening intracranial hemorrhages. FXIII activity testing ultimately revealed a level of less than 5%, confirming severe FXIII deficiency. He was started on FXIII concentrate prophylaxis with no further bleeding events. This case highlights how diagnostic anchoring and inadvertent treatment with cryoprecipitate can mask FXIII deficiency for decades. Furthermore, it underscores the importance of considering FXIII testing in patients with severe or recurrent bleeding and normal standard coagulation studies.

## Introduction

Factor XIII (FXIII) deficiency is a rare inherited coagulopathy, with an estimated prevalence of approximately one per 1-3 million individuals [1]. This disorder is characterized by a propensity for severe spontaneous bleeding, with intracranial hemorrhage (ICH) representing one of the most feared and life-threatening manifestations. ICH occurs in up to one third of patients with severe congenital FXIII deficiency, a rate considerably higher than the pooled ICH incidence of 2.3 per 1,000 person-years reported across all ages in hemophilia (rising to 7.4 per 1,000 person-years in children and young adults) and the approximately 2% prevalence observed in the largest cohort of type 3 von Willebrand disease (VWD) [1-3]. FXIII deficiency may be either acquired (e.g., due to autoantibodies or systemic illness) or congenital; the latter is much more common and typically identifiable through evaluation of family history, age at symptom onset, and clinical context [1,4].

FXIII is unique among the coagulation factors in that it is a transglutaminase rather than a protease. Its best-known function is to crosslink fibrin polymers and to crosslink α2-antiplasmin to fibrin, strengthening and making the clot resistant to fibrinolysis [5]. A critical diagnostic challenge in FXIII deficiency is that routine coagulation screening tests including prothrombin time (PT), activated partial thromboplastin time (aPTT), and international normalized ratio (INR) typically remain within normal limits, as FXIII functions downstream of fibrin formation and is not assessed by these assays [4]. In practical terms, PT and aPTT measure the speed of clot formation, whereas FXIII acts after a fibrin clot has already formed, crosslinking and stabilizing it against premature breakdown; a patient can therefore form a clot at a normal rate that is structurally fragile and prone to delayed or recurrent hemorrhage. This laboratory discordance frequently results in delayed diagnosis and consequently delayed initiation of targeted therapy [1].

To our knowledge, this is the first reported case describing recurrent ICH in a young man whose inherited FXIII deficiency was obscured for years by cryoprecipitate therapy, erroneously administered for a presumed diagnosis of VWD, and serendipitously successful due to therapeutic overlap. Ultimately, recognition of anchoring bias and an understanding of coagulation factor content and half-life in plasma-derived therapies led to suspicion of FXIII deficiency, appropriate empiric therapy, and subsequent diagnostic confirmation by specialized testing.

## Case presentation

A 32-year-old man originally from Nicaragua presented with ICH. His hematologic history dated to approximately age seven, when he sustained forearm trauma complicated by an extensive hematoma requiring surgical fasciotomy. Following this event, he was presumed to have VWD and subsequently managed with intermittent cryoprecipitate infusions. His last cryoprecipitate infusion was four months prior to this described presentation. His family history was notable for similar bleeding tendencies in a sister and in a maternal relative's child; his mother was also reported to have required recurrent infusions for a bleeding disorder prior to her death.

Initial presentation and workup

The patient initially presented to an outside hospital with moderate left thigh pain and hematoma, occurring without significant antecedent trauma. He was discharged with over-the-counter analgesics and a referral to a specialized hemophilia clinic. Three days later, at his hemophilia clinic appointment, laboratory studies were obtained, and a plan to treat with von Willebrand factor (VWF) and Factor VIII products was documented in the medical record, although administration could not be confirmed. The complete blood count was normal. VWF antigen, ristocetin cofactor activity, and Factor VIII activity were all within normal limits. The patient was scheduled for a return visit in 12 days to review these results.

First ICH

**Figure 1:**
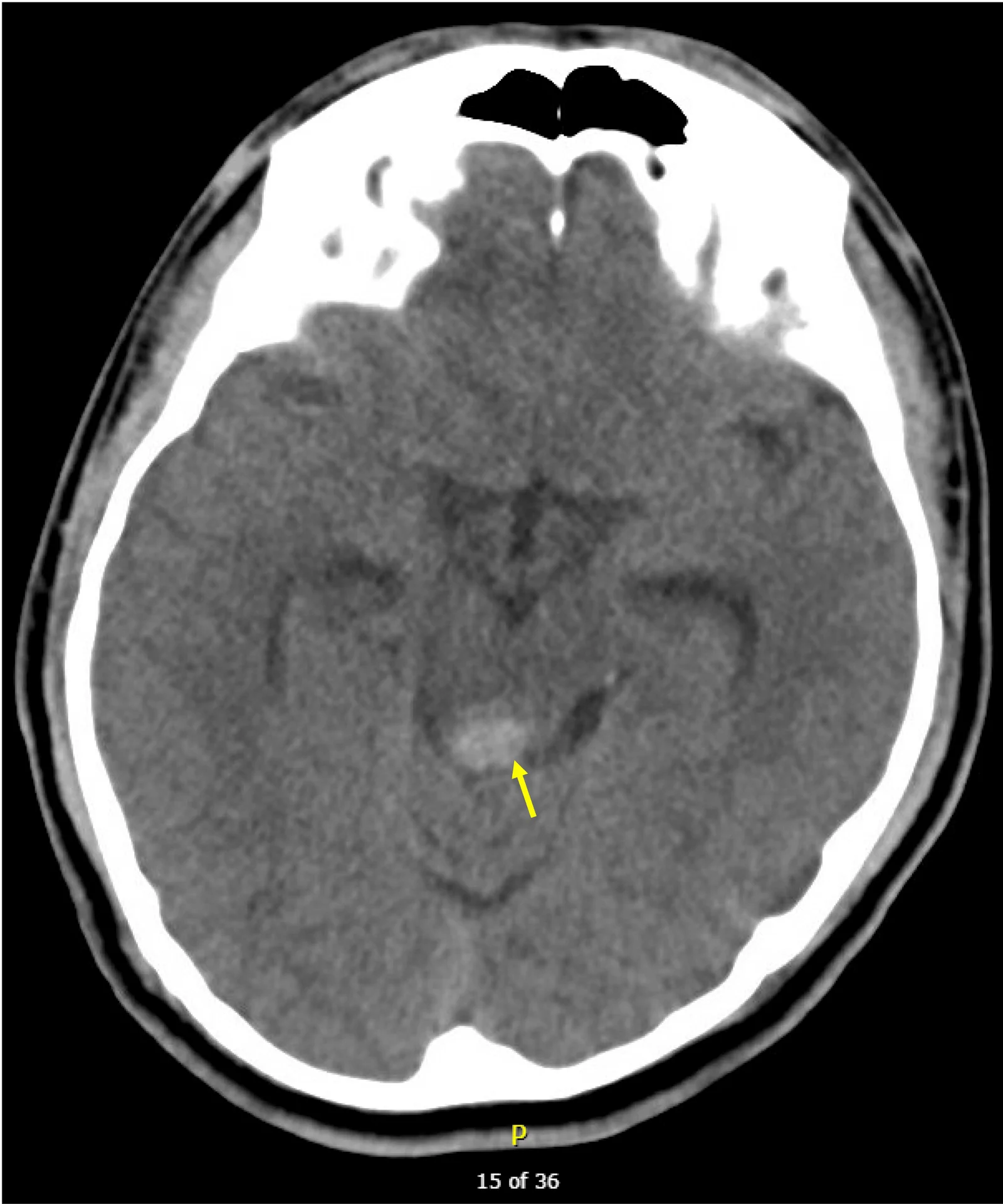
Non-contrast computed tomography (CT) of the brain demonstrating new subarachnoid hemorrhage (SAH) Representative axial slice from the index presentation. Arrow points to the region of the inferior colliculi within the quadrigeminal cistern.

Six days after the hemophilia clinic visit, the patient developed acute severe headaches, blurred vision, diplopia, and left foot numbness, prompting emergency department evaluation. Non-contrast computed tomography (CT) of the brain revealed acute subarachnoid hemorrhage (SAH) centered within the quadrigeminal cistern (Figure 1).

**Figure 2:**
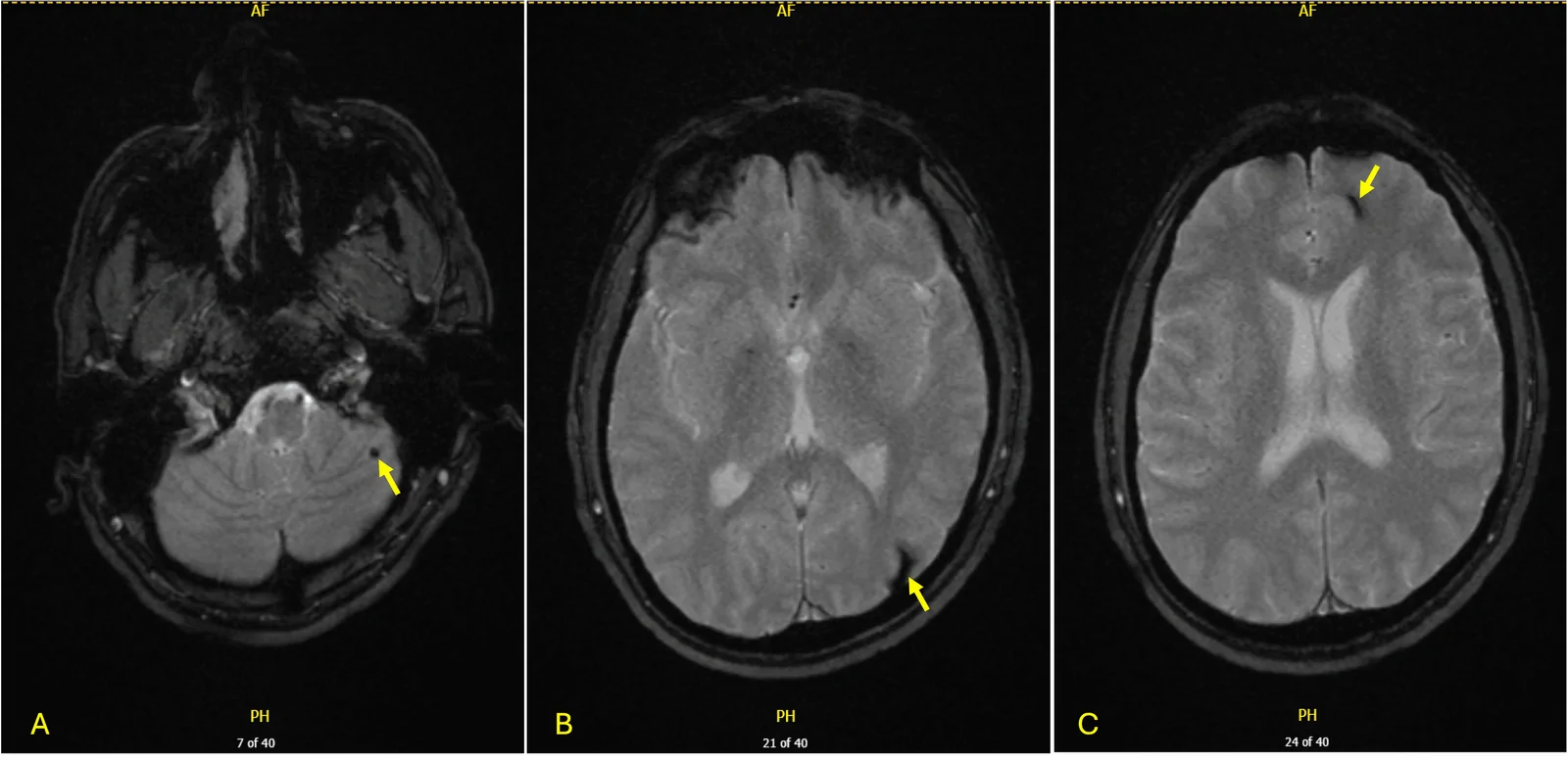
Day 2 MRI brain demonstrating focal susceptibility artifacts suggestive of chronic microhemorrhages Representative axial slices obtained during the first admission. Arrows indicate chronic microhemorrhages in the (A) left cerebellar, (B) left occipital, and (C) left frontal lobes. The multifocal, multilobar distribution of these chronic hemorrhagic lesions, together with the acute SAH in Figure 1, raised early suspicion for an underlying bleeding diathesis rather than a single focal vascular lesion.

During the following 13-day hospitalization, the patient was managed conservatively with Alphanate® (Factor VIII/VWF concentrate) based on the working diagnosis of VWD. Factor VIII and VWF levels drawn throughout hospitalization remained normal, as did routine coagulation studies. On day 2, MRI of the brain showed stable SAH and focal susceptibility artifacts in the left cerebellar (Figure 2A), left occipital (Figure 2B), and left frontal lobes (Figure 2C), suggestive of chronic microhemorrhages.

**Figure 3:**
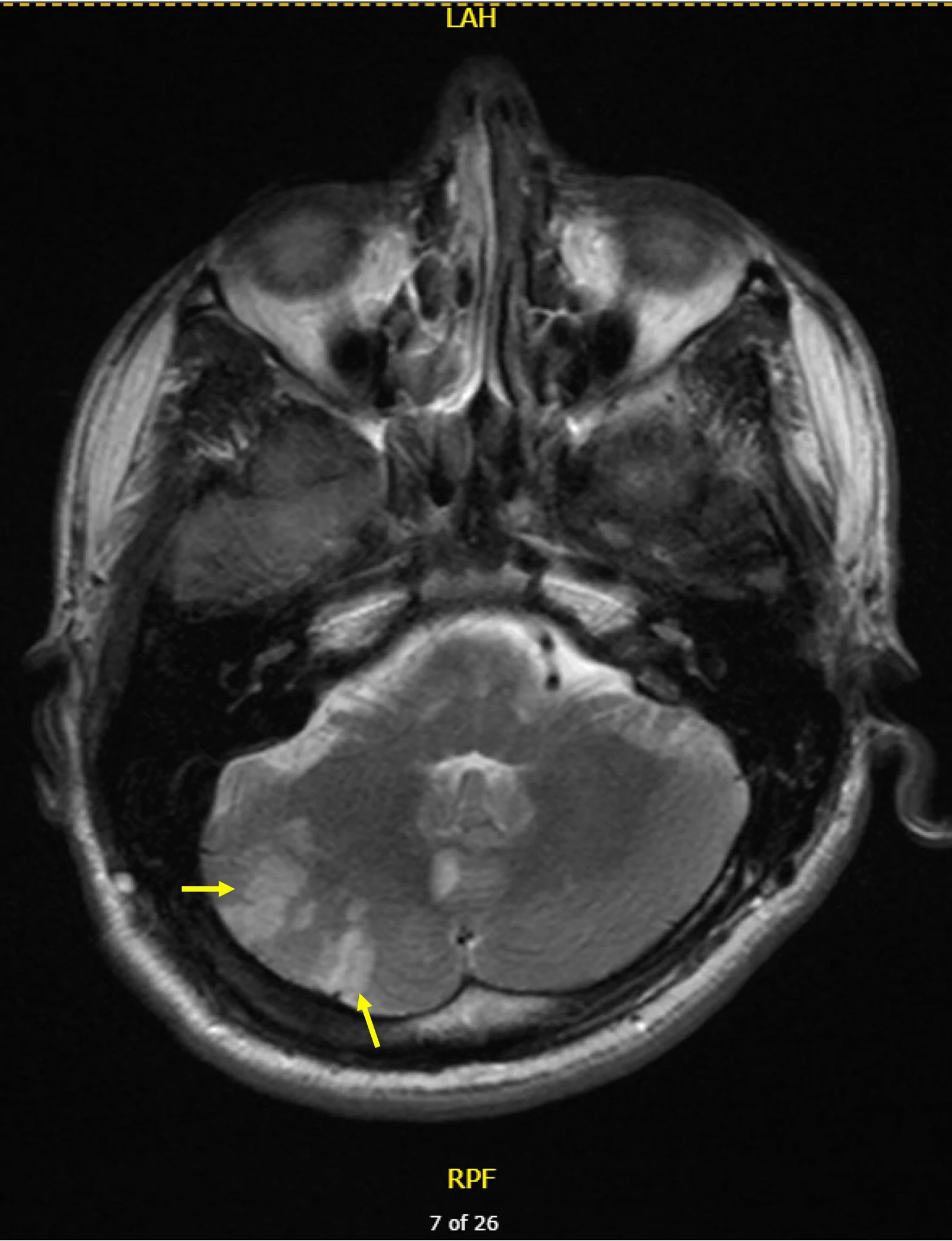
Day 5 MRI brain demonstrating acute infarct in the right cerebellar hemisphere Representative axial slice. Arrows point to areas of acute ischemic infarction within the right cerebellar hemisphere, a distinct process from the hemorrhagic findings in other imaging. This finding prompted a bubble-study echocardiogram and transesophageal echocardiogram, which identified a right-to-left shunt (patent foramen ovale).

Subsequent MRI on day 5 demonstrated multifocal acute small infarcts in the bilateral cerebrum and cerebellum, most notably in the right cerebellar hemisphere (Figure 3). This prompted an echocardiogram with a bubble study, which showed a right-to-left shunt, suggestive of a patent foramen ovale. A transesophageal echocardiogram (TEE) later confirmed this finding.

In the days that followed, the patient's SAH remained stable without radiographic progression; he was discharged with instructions for close outpatient hematology follow-up.

Recurrent hemorrhage and diagnosis

**Figure 4:**
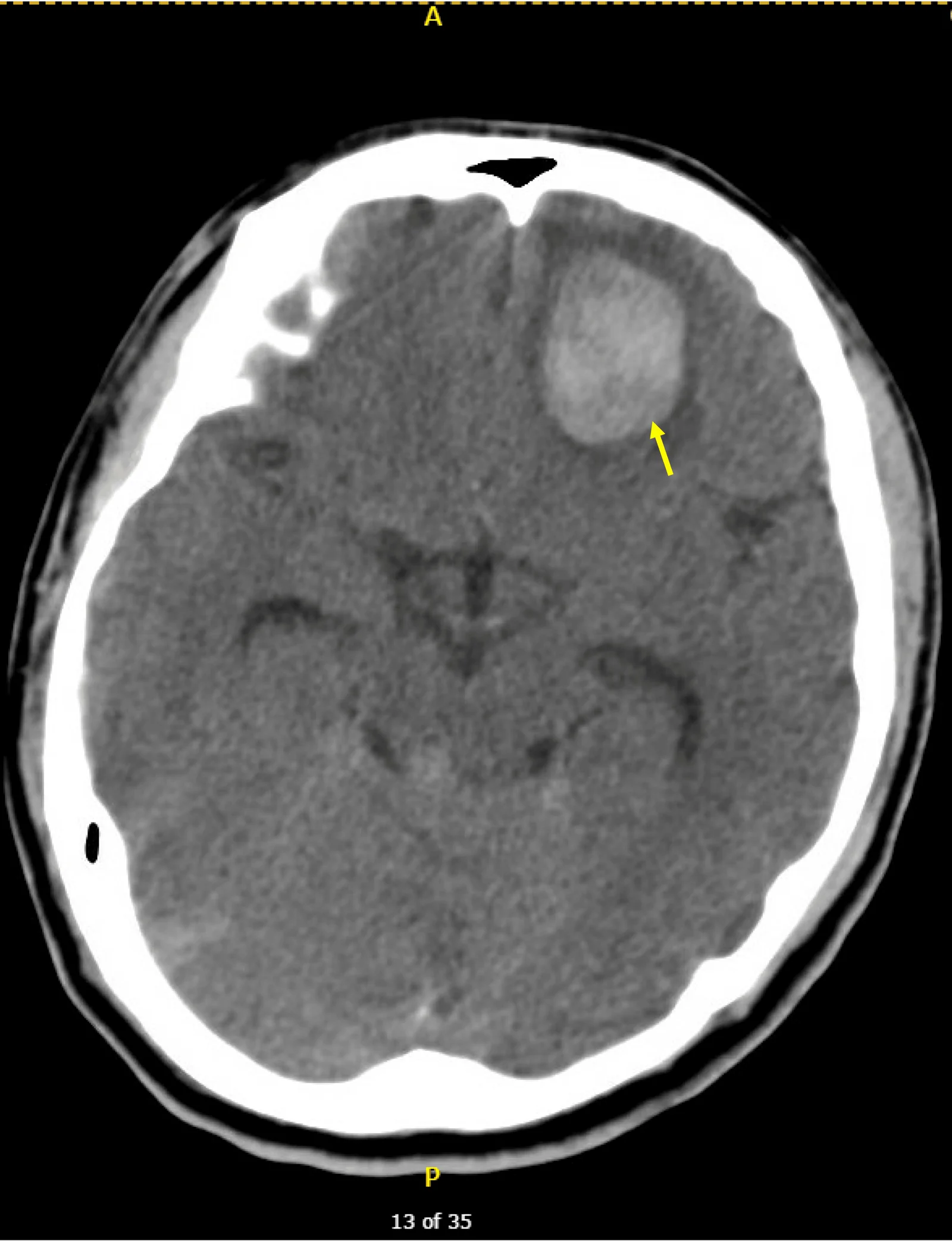
Non-contrast CT of the brain demonstrates new hemorrhage Representative axial slice from the second admission, four days after discharge from the first. Arrow points to a new parenchymal hemorrhage in the superior left frontal lobe; additional new hemorrhages (inferior left frontal lobe, right cerebellum) and new SAH (right parietal convexity) were also identified on this study but are not shown. This recurrence despite a normal aPTT prompted reassessment of the presumed VWD diagnosis and ultimately led to the diagnosis of severe FXIII deficiency. SAH: subarachnoid hemorrhage; aPTT: activated partial thromboplastin time; FXIII: Factor XIII; CT: computed tomography; VWD: von Willebrand disease

However, merely four days after discharge, the patient presented to the emergency department with new acute-onset right-sided hemiparesis and sensory deficits affecting the right face, arm, and leg. Repeat CT imaging revealed new multifocal parenchymal hemorrhages in the superior left frontal lobe, inferior left frontal lobe, and right cerebellum, as well as new acute SAH along the right parietal convexity (Figure 4). He was immediately treated with Alphanate®, although the aPTT just prior to administration was normal. The neurocritical care and hematology services were consulted for evaluation and management.

**Figure 5:**
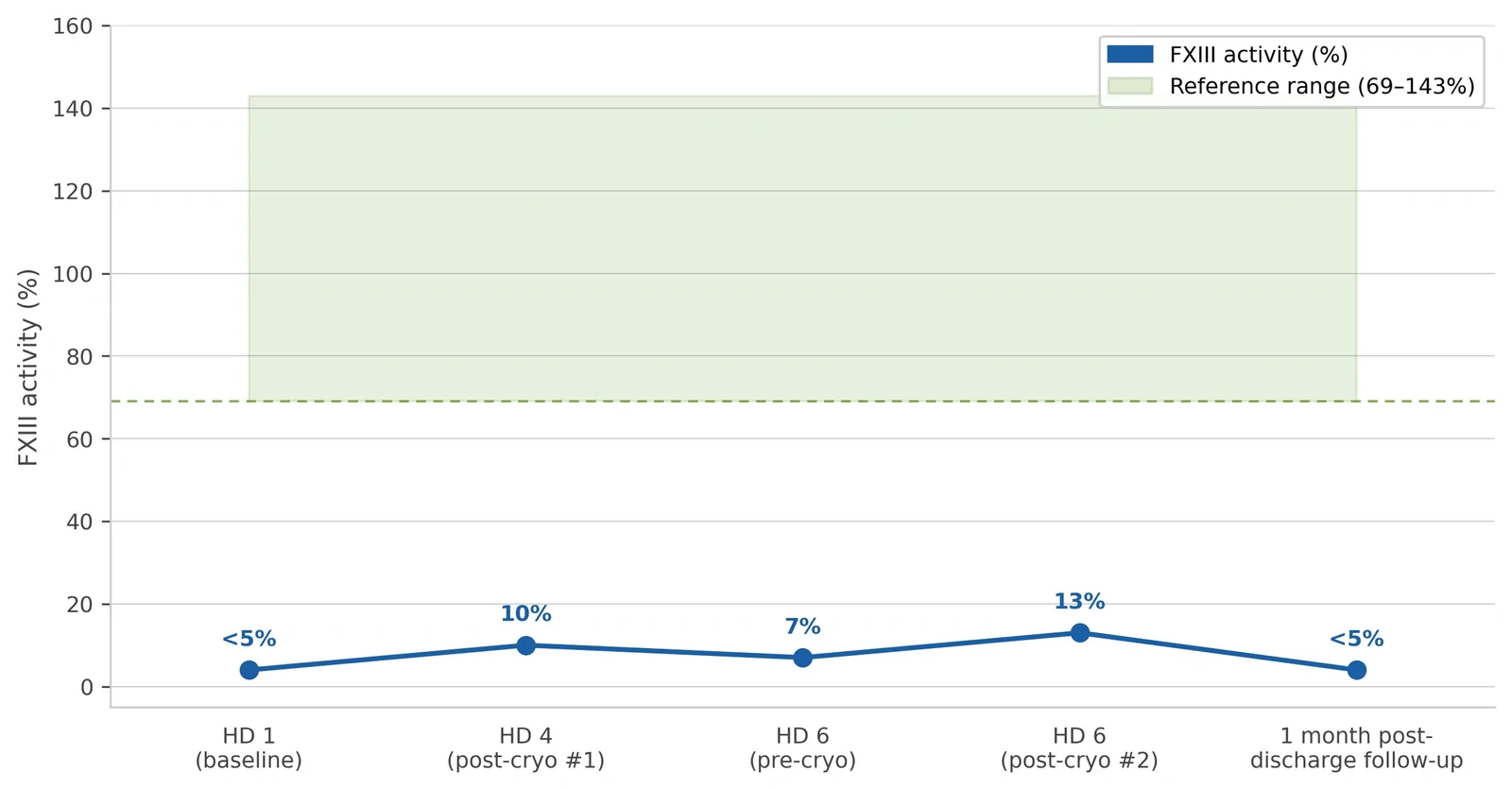
Serial Factor XIII (FXIII) activity measurements during hospitalization and follow-up Values below the lower limit of detection (<5%) are plotted at 4%. The shaded band represents the institutional reference range (69%-143%). HD: hospital day; cryo: cryoprecipitate Figure generated using Python version 3.12.12, with sub-package matplotlib version 3.10.3 (Python Software Foundation, Fredericksburg, VA, USA).

Recurrent ICH with a normal aPTT prompted reassessment of the presumed VWD diagnosis. In the absence of timely specialized testing, management was guided by the patient's long-term response to intermittent cryoprecipitate, suggesting deficiency of a factor with a long half-life not detected by routine assays. Alphanate® was discontinued, and empiric cryoprecipitate and tranexamic acid were initiated while additional studies were pursued. Subsequent factor studies showed elevated Factor VIII and VWF levels, with profoundly decreased FXIII activity identified using the preferred specialized chromogenic assay [6]. Platelet aggregation was also determined to be normal. Serial measurements demonstrated persistently severe FXIII deficiency with expected post-transfusion increases (Figure 5), while PT, INR, and aPTT remained normal throughout.

Treatment and follow-up

**Figure 6:**
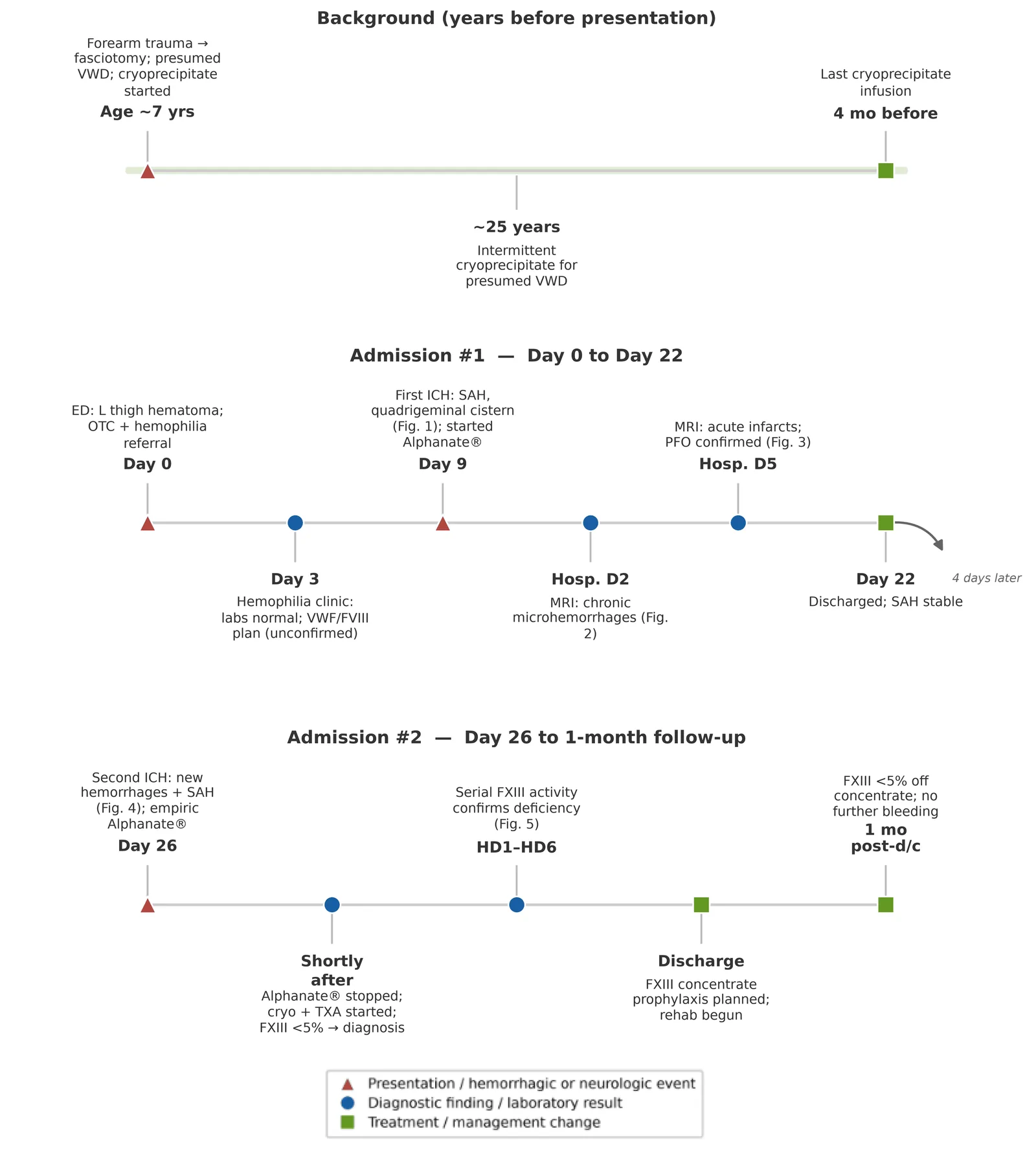
Timeline of presentations, investigations, treatment changes, and diagnosis Timeline of the patient's clinical course, from initial childhood presentation through diagnosis and one-month follow-up. Marker shape and color indicate event category: red triangle: presentation or hemorrhagic/neurologic event; blue circle: diagnostic finding or laboratory result; green square: treatment or management change. Day numbers are calculated from the patient's initial outside-hospital presentation (day 0); hospital day (HD) and admission (Adm.) numbers refer to the specific hospitalization noted. See Case presentation for full clinical details. ED: emergency department; VWD: von Willebrand disease; OTC: over-the-counter; ICH: intracranial hemorrhage; SAH: subarachnoid hemorrhage; PFO: patent foramen ovale; TXA: tranexamic acid; FVIII: Factor VIII; VWF: von Willebrand factor; FXIII: Factor XIII Figure generated using Python version 3.12.12, with sub-package matplotlib version 3.10.3 (Python Software Foundation, Fredericksburg, VA, USA).

While hospitalized, the patient received cryoprecipitate infusions for acute hemostatic stabilization. Long-term prophylactic therapy was subsequently initiated with plasma-derived FXIII concentrate. Tranexamic acid was retained as a rescue antifibrinolytic therapy for breakthrough bleeding events or with procedures. The patient’s neurological deficits have been managed with rehabilitation. No further hemorrhagic events have been documented on prophylaxis (Figure 6).

## Discussion

This case highlights the diagnostic limitations of routine coagulation testing in FXIII deficiency, the risk of diagnostic anchoring on more common bleeding disorders, and the benefit of a more detailed knowledge of blood products in the face of a life-threatening hemorrhagic emergency. Several case reports have described ICH as a presenting or recurrent manifestation of FXIII deficiency, consistently noting that routine coagulation studies remain normal throughout [7-9]. In a notable prior report, a patient presented with thalamic hemorrhage, recovered, then returned six months later with five new foci of spontaneous ICH and ultimately died before FXIII deficiency was identified, underscoring the lethality of delayed diagnosis [7]. Our case shares these hallmark features: recurrent multifocal ICH, a young patient, normal coagulation screening, and a significant delay before FXIII-specific testing was obtained. What distinguishes our case is the mechanism of diagnostic delay: longstanding cryoprecipitate therapy, administered for a misdiagnosed bleeding disorder, contains FXIII in addition to fibrinogen, VWF, and Factor VIII (Table 1) [10,11]. Unlike in the cases reported by Perez et al. [7], Sadiq et al. [8], and Wang et al. [9], in which FXIII deficiency was either never previously treated or diagnosed relatively early in the disease course, in our case, the longstanding cryoprecipitate therapy provided partial FXIII replacement for over two decades and is the most plausible explanation for the masking of the underlying deficiency. Transition to a single-factor concentrate lacking FXIII is temporally and mechanistically consistent with unmasking the severe deficiency and precipitating life-threatening hemorrhage. To our knowledge, this pattern in which cryoprecipitate exposure obscures congenital FXIII deficiency until transition to a targeted single-factor concentrate precipitates life-threatening hemorrhage has not been previously described in the literature (Figure 6).

**Table 1:** Coagulation factor concentrations in FFP versus cryoprecipitate, with corresponding plasma half-lives “+” denotes presence at baseline FFP levels; fold-enrichment values reflect concentration in cryoprecipitate relative to FFP. The FXIII row illustrates the 2-3× enrichment present in cryoprecipitate, a clinically significant source of inadvertent FXIII replacement in this case. “-” denotes minimal or absent enrichment relative to FFP. FFP: fresh frozen plasma; VWF: von Willebrand factor; FXIII: Factor XIII

Factor	FFP factor concentration	Cryoprecipitate factor concentration	Half-life (hours)
Fibrinogen	+	2-3×	96-150
Factor II	+	-	60
Factor V	+	-	24
Factor VII	+	-	4-6
Factor VIII	+	5×	11-12
Factor IX	+	-	22
Factor X	+	-	35
Factor XI	+	-	60
Factor XIII	+	2-3×	144-300
VWF	+	9×	8-12
Others	+	-	—

While FXIII deficiency can occur via both acquired and congenital etiologies, in the case of this patient, an acquired etiology was considered unlikely given the patient's childhood age of onset; absence of a preceding autoimmune, infectious, hepatic, or malignant process; and a multigenerational family history of similar bleeding, all of which favor a congenital process. However, we acknowledge that neither formal exclusion of an acquired process (e.g., FXIII autoantibody testing) nor confirmatory genetic testing was performed, and this is noted as a limitation.

Delayed availability of FXIII activity testing further complicated management, necessitating empiric treatment based on clinical suspicion. Although some institutions may have in-house urea clot solubility testing, experts advise against its use, noting multiple variables leading to poor standardization and an inability to detect all but the most severe deficiencies [6]. This poor sensitivity can be additionally compounded by emergency administration of FXIII-containing plasma products and lead to erroneous rejection of an FXIII deficiency diagnosis if specialized testing is not pursued [12]. Furthermore, even in-house assays require observation of the clot for 24 hours [13].

Early identification of FXIII deficiency is critical, as prophylactic FXIII replacement substantially reduces the risk of recurrent ICH. This case underscores the need to consider FXIII deficiency in patients with severe bleeding and normal coagulation studies, particularly when a previous cryoprecipitate treatment response is present.

## Conclusions

Clinicians should maintain a high index of suspicion for FXIII deficiency in patients presenting with severe or recurrent bleeding complications, particularly ICH, in the setting of persistently normal routine coagulation studies, especially when accompanied by a suggestive family history. Standard coagulation screening (PT, aPTT, and INR) is inadequate for detecting this disorder, and FXIII activity testing is essential for definitive diagnosis. A critical and underrecognized diagnostic pitfall highlighted by this case is that longstanding treatment with cryoprecipitate may inadvertently mask FXIII deficiency; transition to a single factor concentrate that lacks FXIII may precipitate life-threatening hemorrhage. Once identified, timely initiation of prophylactic FXIII concentrate therapy is critical to preventing catastrophic complications.
